# Optimizing Latent Tuberculosis Treatment Strategies Among Immigrants From High-Burden Settings

**DOI:** 10.64898/2026.07.13.26357974

**Published:** 2026-07-16

**Authors:** Alexa Tabackman, Meagan Karoly, Karen R. Jacobson, C. Robert Horsburgh, Benjamin Linas, Jeffrey Campbell, Carlos Acuña-Villaorduña, Pranay Sinha

## Abstract

**Importance:**

Tuberculosis preventive therapy is central to reducing tuberculosis, and foreign-born individuals account for most US tuberculosis cases. Current US Preventive Services Task Force guidance recommends testing and treating all foreign-born individuals regardless of age or time since immigration, yet the risks of disease progression and of treatment-related harm are not uniform across these groups.

**Objective:**

To evaluate the cost-effectiveness and health outcomes of tuberculosis infection treatment strategies among immigrants from high-burden settings, stratified by age and time since immigration.

**Design:**

Decision analytical model using individual-level microsimulation (Markov model) over a 30-year horizon, with deterministic and probabilistic (second-order Monte Carlo) sensitivity analyses. Costs and outcomes were discounted at 3%.

**Setting:**

US federally funded tuberculosis clinic care (healthcare-sector perspective), using observed data from the Boston Medical Center/Boston Public Health Commission tuberculosis clinic and published literature.

**Participants:**

A simulated cohort of 10 000 IGRA-positive, foreign-born adults from high tuberculosis incidence settings (excluding immunosuppressed individuals), modeled as recent or remote (immigrated 25 years earlier) immigrants at ages 35 and 65 years.

**Interventions:**

Rifampin daily for 4 months, isoniazid daily for 9 months, or no preventive therapy.

**Main Outcomes and Measures:**

Costs, disability-adjusted life-years (DALYs), incident tuberculosis cases and deaths, treatment completion, and incremental cost-effectiveness ratios (ICERs), with the proportion of simulations in which each strategy was optimal at a willingness-to-pay threshold of $50 000 per DALY averted.

**Results:**

Among recent immigrants, rifampin was the dominant strategy at ages 35 and 65 years (optimal in 88.5% and 93.9% of simulations), yielding the fewest tuberculosis cases (119.44 and 82.31 per 10 000) and the highest treatment completion (71.4% and 67.7%). Among remote immigrants, rifampin remained the dominant strategy (optimal in 53.41% of simulations), followed by no treatment. In older remote immigrants, no treatment was optimal in 94.7% of simulations. ICERs for treatment vs no treatment were unfavorable ($193 600 and $412 857 per DALY averted for rifampin and isoniazid, respectively, at age 65).

**Conclusions and Relevance:**

In this decision analytical model, rifampin was cost-effective for recent immigrants, whereas no treatment was optimal for older remote immigrants. Age and time since immigration may help risk-stratify tuberculosis infection treatment and reduce unnecessary treatment in lower-risk populations.

**Key Points:**

**Question**

Among immigrants from high–tuberculosis-burden settings with a positive interferon-gamma release assay (IGRA), how do rifampin, isoniazid, and no preventive therapy compare in cost effectiveness across age and time since immigration?

**Findings**

In this decision analytical model of 10 000 simulated immigrants, rifampin was the optimal strategy for recent immigrants at ages 35 and 65 years (optimal in 88.5% and 93.4% of simulations), whereas no treatment was optimal for older immigrants who had immigrated 25 years earlier (94.7% at age 65).

**Meaning**

Age and time since immigration may inform tuberculosis infection treatment decisions and reduce unnecessary treatment in lower-risk immigrants.

## Full Text Availability

The license terms selected by the author(s) for this preprint version do not permit archiving in PMC. The full text is available from the preprint server.

